# The effect of repeated remote ischemic postconditioning after an ischemic stroke (REPOST): A randomized controlled trial

**DOI:** 10.1177/17474930221104710

**Published:** 2022-06-14

**Authors:** Thijs RJ Landman, Yvonne Schoon, Michiel C Warlé, Frederick JA Meijer, Frank-Erik De Leeuw, Dick HJ Thijssen

**Affiliations:** 1 Department of Physiology, Radboud Institute for Health Sciences, Radboud University Medical Center, Nijmegen, The Netherlands; 2 Department of Geriatric Medicine, Radboud Institute for Health Sciences, Radboud University Medical Center, Nijmegen, The Netherlands; 3 Department of Surgery, Radboud University Medical Center, Nijmegen, The Netherlands; 4 Department of Medical Imaging, Radboud University Medical Center, Nijmegen, The Netherlands; 5 Donders Center for Medical Neuroscience, Department of Neurology, Radboud University Medical Center, Nijmegen, The Netherlands

**Keywords:** Acute stroke therapy, MRI, neuroprotection, treatment, ischemic stroke, therapy

## Abstract

**Background and Aims:**

A potential strategy to treat ischemic stroke may be the application of repeated remote ischemic postconditioning (rIPostC). This consists of several cycles of brief periods of limb ischemia followed by reperfusion, which can be applied by inflating a simple blood pressure cuff and subsequently could result in neuroprotection after stroke.

**Methods:**

Adult patients admitted with an ischemic stroke in the past 24 h were randomized 1:1 to repeated rIPostC or sham-conditioning. Repeated rIPostC was performed by inflating a blood pressure cuff around the upper arm (4 × 5 min at 200 mm Hg), which was repeated twice daily during hospitalization with a maximum of 4 days. Primary outcome was infarct size after 4 days or at discharge. Secondary outcomes included the modified Rankin Scale (mRS)-score after 12 weeks and the National Institutes of Health Stroke Scale (NIHSS) at discharge.

**Results:**

The trial was preliminarily stopped after we included 88 of the scheduled 180 patients (average age: 70 years, 68% male) into rIPostC (n = 40) and sham-conditioning (n = 48). Median infarct volume was 2.19 mL in rIPostC group and 5.90 mL in sham-conditioning, which was not significantly different between the two groups (median difference: 3.71; 95% CI: −0.56 to 6.09; p = 0.31). We found no significant shift in the mRS score distribution between groups. The adjusted common odds ratio was 2.09 (95% CI: 0.88–5.00). We found no significant difference in the NIHSS score between groups (median difference: 1.00; 95% CI: −0.99 to 1.40; p = 0.51).

**Conclusion:**

This study found no significant improvement in infarct size or clinical outcome in patients with an acute ischemic stroke who were treated with repeated remote ischemic postconditioning. However, due to a lower-than-expected inclusion rate, no definitive conclusions about the effectiveness of rIPostC can be drawn

## Introduction

A potential new strategy to treat acute ischemic stroke may be the application of remote ischemic postconditioning (rIPostC). This refers to an intervention where an ischemic stimulus is applied distant from the brain (e.g. a limb) within hours after an ischemic stroke, potentially resulting in neuroprotection.^1^ rIPostC consists of several cycles of brief periods of limb ischemia followed by reperfusion, which can be applied by inflating a simple blood pressure cuff.^2–4^ The presumed neuroprotective effects of rIPostC are hypothesized to be related to a reduction of ischemia reperfusion injury in the brain after the ischemic stroke and are supposedly most prominent when rIPostC is started as soon as possible after the onset of symptoms.^5,6^ Several studies support the ability of rIPostC to reduce neural damage after reperfusion.^7,8^ Moreover, it has been postulated that, in addition to the short-lasting benefits of a single bout of rIPostC, longer-lasting benefits may be induced with repeated conditioning,^1^ which has been confirmed in several preclinical studies.^9^ The use of repeated rIPostC may be a simple strategy to minimize the clinical impact of ischemic stroke. Importantly, rIPostC is virtually cost-free, non-pharmacological, non-invasive and without any known adverse effects. This study examined whether adding repeated rIPostC to the current treatment of stroke patients has beneficial effects on infarct size and clinical outcome.

## Aims and hypothesis

In the current randomized controlled trial, we aimed to evaluate the effect of rIPostC on infarct size and clinical outcome in patients presenting to the hospital with an acute ischemic stroke. We hypothesized that repeated rIPostC during the first days following an ischemic stroke reduces infarct size and since infarct size is related to functional recovery,^10^ repeated rIPostC could potentially also minimize the clinical impact of an ischemic stroke.

## Methods

The REPOST (The effect of REpeated rIPostC on infarct size in patients with an ischemic STroke) was a randomized single-blind placebo-controlled clinical trial, performed at a single center (Radboud University Medical Center (Radboudumc)) in Nijmegen, The Netherlands. The study was approved by the relevant ethical committee (CMO Arnhem-Nijmegen, Registration No. 2017-3711). The protocol of this RCT was described in detail in a previous article^11^ and registered at “Netherlands Trial Register” (NTR6880). In this article, we will only summarize the most important parts of the protocol.

## Participants

Patients aged 18 years or older with an ischemic stroke in the past 24 h who were admitted to the Radboudumc were eligible for inclusion. Exclusion criteria were unstable vital signs or contra-indications for either rIPostC (upper extremity injury or bilateral mastectomy) or MRI (e.g. pacemaker, vascular clips, cochlear implants, or other implanted metal objects).

An oral assent was obtained for all participants to be able to start the intervention as soon as possible after the onset of stroke. All participants provided written informed consent within 48 h after oral assent. Participants who received a change in diagnosis during hospitalization, established by a neurologist were excluded from the analysis.

## Randomization and intervention

Participants were randomized in a 1:1 ratio to either rIPostC or sham-conditioning. Stratification was performed for the revascularization treatment received (i.e. intravenous thrombolysis, thrombectomy, or no revascularization treatment). For the intervention, a manual blood pressure cuff was inflated around the non-paretic upper arm by one of the researchers. All participants received four cycles of 5-min inflation of the blood pressure cuff, followed by 5 min of deflation. This procedure was repeated twice daily (morning and afternoon) with at least 6 h in between and continued for the duration of hospitalization with a maximum of 4 days. The level of cuff inflation differed between the groups. In the rIPostC group, the cuff was inflated to 200 mm Hg (or 20 mm Hg above systolic blood pressure, if systolic blood pressure was > 180 mm Hg), mediating full blockage of arterial blood flow. In the sham-conditioning group, the cuff was inflated to 50 mm Hg (or 10 mm Hg below diastolic blood pressure, if diastolic blood pressure was < 60 mm Hg) which did not induce any ischemia.

## Primary outcome measure

The primary outcome was infarct size on Day 4 after admission (or at discharge if discharge was before Day 4). The infarct size was evaluated by the brain MRI diffusion-weighted imaging (DWI) using a 1.5 Tesla MRI scanner (Siemens^®^ Avanto). The infarct size was manually annotated and analyzed by a trained researcher. All annotated DWI areas were checked by a neuroradiologist. To ensure blind analyses, all MRI images were blinded by an independent researcher prior to analysis and unblinded after the completion of the trial.

## Secondary outcome measures

As a secondary outcome, the modified Rankin scale (mRS) score^12–14^ was used to determine clinical outcome after 12 weeks. To assess clinical outcome at the end of hospitalization, the National Institutes of Health Stroke Scale (NIHSS)^15^ was used. Both of these scores were assessed by a (blinded) clinical physician or nurse from the neurology department.

## Statistical analyses

Analyses were performed using RStudio.^16^ All data were analyzed according to an intention-to-treat analysis for all included patients. Continuous variables were checked for normality and analyzed with an independent t-test or a non-parametric alternative when the data were not normally distributed (Mann–Whitney U test). To analyze dichotomous variables, a chi-square test was used. The difference in infarct size between the rIPostC and sham-conditioning group was analyzed using a Mann–Whitney U test and presented with a 95% CI for differences between both groups. This analysis was done on the full data, and a pre-determined sub-analysis was performed without the patients who did not have a visible infarct on the MRI at Day 4 (or discharge). To analyze the mRS score, the odds ratio for a shift in the direction of a better outcome on the mRS was assessed in both groups.^17^ This ratio was estimated with ordinal logistic regression and was calculated for all possible cut-off values on the mRS.

## Sample size calculation

As described in our protocol article,^11^ the sample size was estimated based on two trials with a similar stroke population.^18,19^ We expected a clinically relevant difference of 15 mL in infarct size between the two randomized groups. We estimated a standard deviation of 36 cm^3^ based on the previous trials. With an α = 0.05 and a power β = 0.80, we calculated that we needed n = 90 per study arm. Including an expected dropout rate of 10%, we aimed to include 100 patients in each group.

## Results

**Figure 1.**
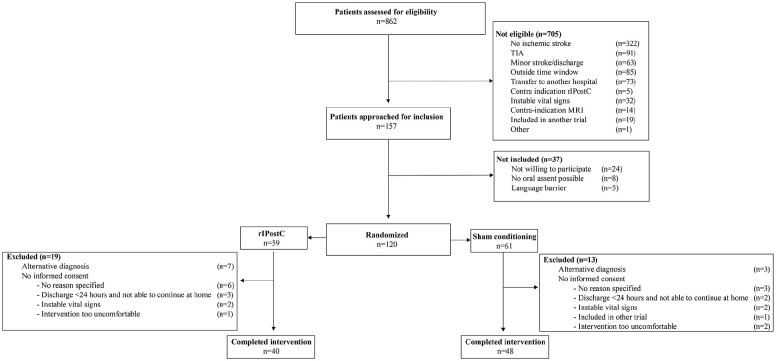
Flowchart REPOST trial.

The trial was preliminarily stopped at October 24, 2021 because of too low inclusion rates. Between April 2018 and October 2021, a total of 862 patients were assessed for eligibility. However, 157 patients were eligible of which 120 were randomized after oral assent; 59 in the rIPostC group, and 61 in the sham-conditioning group. Some participants were excluded after randomization, resulting in a total of 40 patients in the rIPostC group and 48 in the sham-conditioning group who can be used for the final analysis. The reasons for exclusion after randomization were an alternative diagnosis (n = 10) or the inability to obtain informed consent (n = 22). The flowchart is presented in Figure 1. The participants had an average age of 70 years, and 68% was male. All baseline characteristics are presented in Table 1.

**Table 1.** Baseline characteristics REPOST.

Characteristics	rIPostC (N = 40)	Sham (N = 48)
Age (years)	72.3 (±8.9)	67.4 (±12.9)
Sex (male sex)	28 (70.0)	32 (66.7)
BMI (m/kg^2^)	26.1 (±3.7)	27.0 (±4.4)
Blood pressure (mm Hg)		
Systolic	147 (±20)	142 (±22)
Diastolic	78 (±15)	77 (±15)
Medical history		
Atrial fibrillation	8 (20)	5 (10.4)
Hypertension	21 (52.5)	22 (45.8)
TIA	1 (2.5)	6 (12.5)
Stroke	7 (17.5)	10 (20.8)
Myocardial infarction	10 (25.0)	15 (31.3)
Diabetes mellitus	2 (5.0)	9 (18.8)
Alcohol use	31 (77.5)	33 (68.8)
Smoking status		
Never	10 (25.0)	16 (33.3)
Former	21 (52.5)	23 (47.9)
Active	9 (22.5)	9 (18.8)
NIHSS score baseline revascularization treatment received	5.0 [0, 25]	6.5 [0, 28]
Intravenous thrombolysis	23 (57.5)	27 (56.3)
Intra-arterial thrombectomy	10 (25.0)	12 (25.0)
Time from stroke onset to start rIPostC (h)	11.8 (±5.9)	14.1 (±6.6)
Time from stroke onset to presentation (h)	3.5 (±3.8)	4.1 (±4.9)
Cycles of rIPostC/sham-conditioning received	4 [2, 9]	4 [2, 8]

BMI: body mass index, TIA: transient ischemic attack, NIHSS: National Institutes of Health Stroke Scale; rIPostC: remote ischemic postconditioning.

Data are reported as a mean (± SD), median [min, max], or n (%). Alcohol use was defined as drinking any alcohol containing beverages within the last few weeks.

## The effect of rIPostC on infarct size

**Figure 2.**
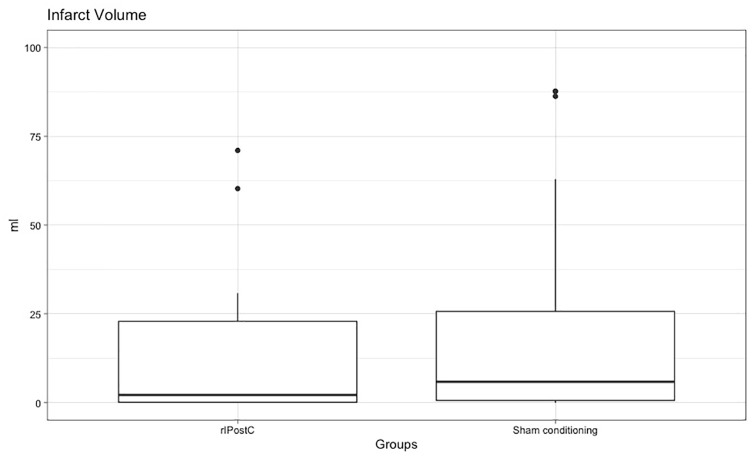
The effect of rIPostC on infarct volume. rIPostC: remote ischemic postconditioning; mL: milliliters. A boxplot that represents the infarct volume (in milliliters) for the sham-conditioning and rIPostC group at discharge. There were four patients with an infarct volume > 100 mL that are not visible in this graph but were used to determine the other characteristics in the boxplot.

MRI data were available for 81 patients (35 in the rIPostC group and 46 in the sham-conditioning group) because it was not possible to schedule the MRI for 7 patients due to logistical issues (MRI fully booked (n = 5) and MRI broken (n = 2)). The mean time between the onset of stroke and the MRI was 56.7 h (rIPostC: 57.8 ± 28.6; sham: 55.8 ± 24.0). The median infarct volume was 2.19 mL in the rIPostC group and 5.90 mL in the sham-conditioning group, which was not significantly different between the two groups (median difference: 3.71; 95% CI: −0.56 to 6.09; p = 0.31; Figure 2).

After exclusion of 16 patients that had no visible infarct on the MRI, we performed a sub-analysis on the 65 patients with a visible DWI lesion (27 in the rIPostC group and 38 in the sham-conditioning group). The median infarct volume was 3.84 mL in the rIPostC group and 11.60 mL in the sham-conditioning group, which was also not significantly different between the two groups (median effect: 7.76; 95% CI: −1.82 to 10.21; p = 0.29).

## The effect of rIPostC on clinical outcome

**Figure 3.**
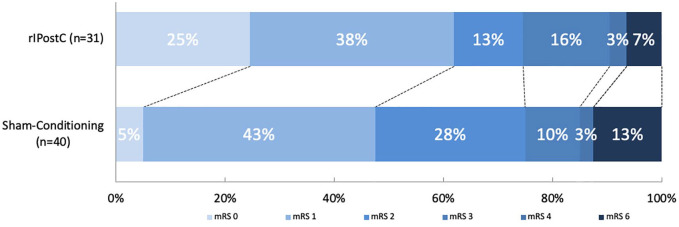
The effect of rIPostC on the mRS score. The distribution of the mRS scores for the rIPostC and sham-conditioning group after 12 weeks. There were no patients with an mRS score of 5.

The mRS score was available for 72 patients after 12 weeks (32 in the rIPostC group and 40 in the sham-conditioning group). We found no significant shift in the distribution of the mRS score in favor of rIPostC. The adjusted common odds ratio was 2.09 (95% CI: 0.88–5.00; Figure 3). There was no absolute between-group difference in the proportion of patients who were functionally independent (mRS score < 3; 75% in the rIPostC group and 75% in the sham-conditioning group; χ^2^ = 0.00; p = 1.00).

The NIHSS score at discharge was available for 83 patients (38 in the rIPostC group and 45 in the sham-conditioning group). The median NIHSS score was 1 in the rIPostC group and 2 in the sham-conditioning group, which was not significantly different between the two groups (median difference: 1.0; 95% CI: −0.99 to 1.40; p = 0.51). No participants died before discharge from the neurology ward.

## Feasibility, tolerability, and safety

The mean time from the onset of symptoms to the start of the intervention was 13.0 h (rIPostC: 11.8 ± 5.9; sham: 14.1 ± 6.6). The patients in both groups received a median of four cycles, with a minimum of two and a maximum of nine (in the rIPostC group) and eight (in the sham-conditioning group) cycles. However, 3 of the 120 randomized patients dropped out because of intolerability for the intervention (i.e. general burden too high or blood pressure cuff too painful). Remarkably, two of these participants were randomized in the sham-conditioning group. In total, 347 cycles of rIPostC and sham-conditioning were administered in the 88 patients who completed the intervention. In total, 343 cycles were completed per protocol (98.8%); 160 in the rIPostC group (97.5%) and 183 in the sham-conditioning group (100%). From the four preliminary ended cycles, one cycle was preliminary terminated after 3 × 5 min due to intolerability, but not severe enough to cause dropout from the study of this participant. The other three cycles were not performed per protocol, as a longer rest interval was taken between two subsequent cuff inflations because of a toilet visit of the patient (10-min interval instead of 5 min). There were no procedure-related adverse events.

## Discussion

We aimed to investigate the effect of repeated rIPostC in ischemic stroke patients. We were not able to demonstrate that patients with an acute ischemic stroke significantly benefit from repeated rIPostC during hospitalization. Specifically, treatment with repeated rIPostC did not significantly reduce infarct size or improve clinical outcome. This absent effect of rIPostC might be related to a smaller than planned sample size, resulting in a type II error.

Our finding that *repeated* rIPostC could not demonstrate to improve infarct size in the brain after an ischemic stroke is in line with other studies that investigated the effect of *single* per- and postconditioning in this patient population.^7,20^ Hougaard et al.^7^ showed that single remote ischemic conditioning (RIC) performed during revascularization treatment (perconditioning) had no impact on penumbral salvage, final infarct size, and infarct growth after 1 month. In a recently published study, Pico et al.^20^ demonstrated that remote ischemic perconditioning, initiated within 6 h after onset of stroke symptoms, did not reduce infarct growth compared to sham-conditioning. Although this last study was designed as proof of concept, they rejected the hypothesis that a single session of remote ischemic perconditioning has a clinically meaningful effect. More details on other randomized controlled trials investigating the effect of RIC in stroke patients can be found in Supplemental Table 1.

We note that our findings are not in line with the findings of preclinical studies that showed promising results of the application of repeated rIPostC in rodent models.^21,22^ This translational gap between preclinical and clinical studies is a recurring issue for research into the effects of RIC. It may be partly due to the characteristics of the ischemic lesion (e.g. mechanically induced in animals versus spontaneous in humans) but also heterogeneity in humans as opposed to homogeneous animals in preclinical studies.^23^ For example, older age seems associated with an attenuated efficacy of conditioning stimuli. In our study, we found a baseline difference in age between the groups, with the rIPostC group being almost 5 years older than the sham-conditioning group. There also was a baseline difference in NIHSS score. These differences may have influenced the results. We explored, using a multivariate regression analysis, whether age and baseline NIHSS were related to infarct size. We found no significant relation between age and baseline NIHSS with our primary outcome.

We are aware that the patients in our study had relatively small infarct sizes and 19% of the patients had no visible infarcts on the MRI at all. This is probably caused by selection bias due to the fact that it was more difficult to get informed consent from patients who were severely affected (or from their family). In our initial sample size calculation, we estimated that a difference of 15 mL in infarct size between the two randomized groups should be considered clinically relevant.^11^ With a median infarct size of 2.19 in the rIPostC group and 5.90 in the sham-conditioning group, an estimated effect size of 15 mL far exceeds the potential difference between the groups. Even in our sub-analysis where we excluded all patients that had no visible infarct size on the MRI, the infarct volumes in the remaining 65 patients were relatively small in both the rIPostC and sham-conditioning group. Although this selection bias is a common phenomenon in stroke research,^24^ the results of our study can therefore only be extrapolated to patients with relatively small infarct volumes.

Although our study was not powered to detect differences in clinical outcome (i.e. mRS score and NIHSS score), we did not find an indication that repeated rIPostC could improve clinical outcome after stroke. Currently, no previous studies have been published that were sufficiently powered to investigate the effect of (single or repeated) rIPostC on clinical outcome after strokes. However, there are some studies that looked at the effect of remote ischemic per- and postconditioning on mRS and NIHSS as secondary outcome measures. Results from these studies are contradictory (Supplemental Table 1). Our results are in line with two studies that also showed no significant improvement in clinical outcome after stroke.^7,25^ For instance, England et al. not only investigated feasibility of both single and repeated rIPostC but also assessed clinical outcome as a secondary outcome and found no improvement in mRS and NIHSS after rIPostC,^25^ but another small pilot trial of this same group did find a significant (although marginal) improvement in the NIHSS score when patients were treated with single rIPostC.^8^ In contrast to these results, two studies by Meng et al.^26,27^ showed a marked improvement in clinical outcome, measured with the mRS score after 180 and 300 consecutive days of repeated rIPostC. In line with our observation that the average infarct size in our population was relatively small, also the clinical outcome in our population favorable in general; the median NIHSS score at the end of hospitalization was only 2%, and 75% of patients were functionally independent (mRS < 3). Even compared with the before-mentioned studies investigating remote ischemic per- and postconditioning in similar patient populations, the people in our study recovered very well from their stroke, both in the rIPostC group and sham-conditioning group. This makes it difficult to detect potentially smaller differences in the mRS score and NIHSS score and therefore we cannot rule out the possibility that rIPostC may be effective for patients with more severe ischemic strokes who have more room for clinical improvement.

### Strengths and limitations

Although we performed a randomized controlled trial with blinded assessment of our outcome measures and achieved a high compliance rate to the interventions, there are also some important limitations that need to be addressed. First, this study was preliminarily stopped before we reached the calculated sample size, which reduces the power to detect potential differences in our outcome measures. It is important to note that some outcome measures were missing due to logistical issues, such as the inability to perform the MRI scan at discharge and lost to follow-up, leading to missing data in our mRS score. Also, a small number of the NIHSS scores were missing (5%), which seems to be mainly attributed to the fact that patients recovered well and were discharged early. Although these data seem to be missing at random and are evenly distributed among the two groups, this could have led to a bias in our results.

Second, in the trial design, we chose absolute difference in infarct size between the two groups at the end of hospitalization as our primary outcome measure. However, we do not have MRI data available on baseline and therefore, we were not able to detect any potential differences in infarct size between the groups on baseline. In addition, an available MRI scan at baseline would also have allowed us to assess infarct growth between admission and discharge from the hospital, which would have given our study more power to detect differences between rIPostC and sham-conditioning.

In conclusion, we found no significant improvement in infarct size or clinical outcome in patients with an acute ischemic stroke who were treated with repeated rIPostC. However, due to a lower-than-expected inclusion rate, no definitive conclusions about the effectiveness of rIPostC can be drawn.

## Supplemental Material

sj-docx-1-wso-10.1177_17474930221104710 – Supplemental material for The effect of repeated remote ischemic postconditioning after an ischemic stroke (REPOST): A randomized controlled trial Click here for additional data file. Supplemental material, sj-docx-1-wso-10.1177_17474930221104710 for The effect of repeated remote ischemic postconditioning after an ischemic stroke (REPOST): A randomized controlled trial by Thijs RJ Landman, Yvonne Schoon, Michiel C Warlé, Frederick JA Meijer, Frank-Erik De Leeuw and Dick HJ Thijssen in International Journal of Stroke
