# Supplementary material for: The effect of repeated remote ischemic postconditioning after an ischemic stroke (REPOST): A randomized controlled trial
Source: Int J Stroke. 2022 Jun 14;18(3):296–303. doi: 10.1177/17474930221104710 (PMC9941800; doi:10.1177/17474930221104710)
Supplement: sj-docx-1-wso-10.1177_17474930221104710 – Supplemental material for The effect of repeated remote ischemic postconditioning after an ischemic stroke (REPOST): A randomized controlled trial [file sj-docx-1-wso-10.1177_17474930221104710.docx]

**Supplemental table 1.** **Summarized description of clinical studies into the effect of remote ischemic (per- and post)conditioning in stroke patients**

| **Study** | **Patients** | **Randomization groups** | **Location of RIC** | **Cycles (occlusion/**  **reperfusion)** | **Baseline NIHSS (median or mean)** | **Time of RIC** | **Effect on infarct size** | **Effect on neurological outcome** |
| --- | --- | --- | --- | --- | --- | --- | --- | --- |
| Meng et al. 2012.^[1]^ | Patients with intracranial arterial stenosis (N=68). | 1: RIC (N=38)  2: Standard treatment only (N=30) | Bilateral upper arm (automated device) | 5x5 min. | RIC: 11  Control: 12 | -Within 30 days after stroke  -Twice daily for 300 consecutive days | N/A | Lower stroke recurrency and improved recovery in mRS |
| Hougaard et al. 2014.^[2]^ | Patients suspected of an ischemic stroke (N=443). | 1: RIC (N=247)  2: Standard treatment (N=196) | Upper limb | 4x5 min. | RIC: 4  Control: 5 | During transportation to the hospital (once) | No effect on penumbral salvage or infarct size.  Lower tissue risk of infarction | No effect on mRS at 90 days |
| Meng et al. 2015.^[3]^ | Patients with intracranial arterial stenosis (N=58). | 1: RIC (N=30)  2: Sham (N=28) | Bilateral upper arm (automated device) | 5x5 min. | RIC: 11  Control: 11 | - Within 7 days after an ischemic stroke or TIA.  - Twice daily for 180 consecutive days | N/A | Lower stroke recurrency. Improved NIHSS and mRS |
| England et al. 2017.^[4]^ | Patients with acute ischemic stroke (N=26). | 1: RIC (N=13)  2: Sham (N=13) | Upper arm | 4x5 min. | RIC: 6  Control: 5 | Within 24 hours after onset of stroke symptoms. | N/A | Improved NIHSS |
| England et al. 2019.^[5]^ | Patients with acute ischemic stroke (N=60) | 1: RIC (N=31)  2: Sham (N=29) | Upper limb | 4x5 min. | RIC: 6  Control: 7 | -Within 6 hours after symptom onset  - Twice daily for 4 days (20 patients) | N/A | No effect (NIHSS at 4 days and mRS at 90 days) |
| Pico et al.2020.^[6]^ | Patients with acute ischemic stroke (N=188) | 1: RIC (N=93)  2: Sham (N=95) | Lower limb (automated device) | 4x5 min. | RIC: 9  Control: 10 | Once within 6 hours of onset | No significant effect on infarct growth | No significant effect on mRS at 90 days |
| He et al. 2020.^[7]^ | Patients with acute ischemic stroke who got IVT (N=49) | 1: RIC (N=24)  2: Sham (N=25) | Upper limb | 4x5 min. | RIC: 6.5  Control: 5 | Twice within 6-24 hours after IVT | N/A | No difference in mRS at 90 days and NIHSS at 1, 7 and 30 days. |
| An et al. 2020.^[8]^ | Patients with acute ischemic stroke who got IVT (N=68) | 1: RIC (N=34)  2: Sham (N=34) | Bilateral upper limb (automated device) | 5x 3 min. | RIC: 6.5  Control: 4.5 | - Within 3 hours of IVT  - Twice daily during hospital stay | N/A | Improved mRS and NIHSS scores at 90 days |
| Che et al. 2019.^[9]^ | Patients with acute ischemic stroke who got IVT (N=30) | 1: RIC (N=15)  2: Sham (N=15) | Bilateral upper limb (automated device) | 5x5 min. | RIC: 7  Control: 5 | - Within 2 hours after IVT  - Twice daily for 7 days | N/A | Improved NIHSS at 30 days. No effect on NIHSS and mRS at 90 days. |

**References**

1. Meng, R., et al., *Upper limb ischemic preconditioning prevents recurrent stroke in intracranial arterial stenosis.* Neurology, 2012. **79**(18): p. 1853-61.

2. Hougaard, K.D., et al., *Remote ischemic perconditioning as an adjunct therapy to thrombolysis in patients with acute ischemic stroke: a randomized trial.* Stroke, 2014. **45**(1): p. 159-67.

3. Meng, R., et al., *Ischemic Conditioning Is Safe and Effective for Octo- and Nonagenarians in Stroke Prevention and Treatment.* Neurotherapeutics, 2015. **12**(3): p. 667-77.

4. England, T.J., et al., *RECAST (Remote Ischemic Conditioning After Stroke Trial): A Pilot Randomized Placebo Controlled Phase II Trial in Acute Ischemic Stroke.* Stroke, 2017. **48**(5): p. 1412-1415.

5. England, T.J., et al., *Remote Ischemic Conditioning After Stroke Trial 2: A Phase IIb Randomized Controlled Trial in Hyperacute Stroke.* J Am Heart Assoc, 2019. **8**(23): p. e013572.

6. Pico, F., et al., *Effect of In-Hospital Remote Ischemic Perconditioning on Brain Infarction Growth and Clinical Outcomes in Patients With Acute Ischemic Stroke: The RESCUE BRAIN Randomized Clinical Trial.* JAMA Neurol, 2020. **77**(6): p. 725-734.

7. He, Y.D., et al., *Remote ischemic conditioning combined with intravenous thrombolysis for acute ischemic stroke.* Ann Clin Transl Neurol, 2020. **7**(6): p. 972-979.

8. An, J.Q., et al., *Safety and efficacy of remote ischemic postconditioning after thrombolysis in patients with stroke.* Neurology, 2020. **95**(24): p. e3355-e3363.

9. Che, R., et al., *rt-PA with remote ischemic postconditioning for acute ischemic stroke.* Ann Clin Transl Neurol, 2019. **6**(2): p. 364-372.
